# Syncope associated with supraventricular tachycardia: Diagnostic role of implantable loop recorders

**DOI:** 10.1111/anec.12850

**Published:** 2021-05-06

**Authors:** Stergios Soulaidopoulos, Petros Arsenos, Ioannis Doundoulakis, Dimitrios Tsiachris, Christos‐Konstantinos Antoniou, Polychronis Dilaveris, Nikolaos Fragakis, Melani Sotiriadou, Skevos Sideris, Athanasios Kordalis, Ageliki Laina, Dimitrios Tousoulis, Konstantinos Tsioufis, Konstantinos A. Gatzoulis

**Affiliations:** ^1^ First Department of Cardiology School of Medicine Hippokration General Hospital National and Kapodistrian University of Athens Athens Greece; ^2^ Athens Heart Centre Athens Greece; ^3^ Third Department of Cardiology Aristotle University of Thessaloniki Thessaloniki Greece; ^4^ State Department of Cardiology Hippokration General Hospital Athens Greece

**Keywords:** electrophysiology test, implantable loop recorder, supraventricular tachycardia, syncope, tilt‐table test

## Abstract

Syncope represents a relatively uncommon symptom of supraventricular tachycardia (SVT). It is likely that an impaired autonomic vasomotor response to the hemodynamic stress of tachycardia is the determinant of hemodynamic changes leading to cerebral hypoperfusion and syncope. In this regard, tilt‐table test may detect abnormalities in the autonomic nervous function and predict the occurrence of syncope during SVT. Electrophysiology studies may reproduce the SVT, distinguish it from other life‐threatening ventricular tachyarrhythmias, and exclude other causes of syncope. Not infrequently mixed syncope mechanisms are revealed during the above diagnostic workup raising doubts about the operating mechanism in the clinical setting. In such cases of uncertainty, an implantable loop recorder, providing long‐term cardiac monitoring, may play a pivotal role in the establishment of the diagnosis, confirming the association of an arrhythmic event with the symptom. Herein, we present four such cases with recurrent unexplained syncope finally attributed to paroxysmal SVT guiding them to a potentially radical treatment through radiofrequency catheter ablation.

## 1 INTRODUCTION

Syncope is a common clinical syndrome, defined as the sudden and temporary loss of consciousness with immediate, spontaneous recovery (Shen et al., 2017). It is the result of inadequate, self‐limited cerebral hypoperfusion, due to a transient drop in systemic blood pressure of brief duration. Depending on the principal pathogenetic mechanism, syncope can be classified as cardiac, reflex, and syncope due to orthostatic hypotension, while several neurological conditions may mimic this event (Saklani et al., 2013). Vasovagal events in the absence of significant underlying cardiac disorders, and cardiac causes, including tachyarrhythmia or bradyarrhythmia with or without structural heart disease, are listed among the most common causes of syncope and present varying prognosis (Soteriades et al., 2002). The identification of the precise mechanism of syncope is crucial to assess prognosis and proceed to the optimal treatment option in order to minimize the risk of recurrence. However, not uncommonly, several different syncope mechanisms may operate in the same patient as suggested by different aspects of the diagnostic approach, namely the electrophysiology study (EPS), the tilt‐table testing (TTT), and the implantable loop recorder (ILR) monitoring (Dilaveris et al., 2020; Gatzoulis, Georgopoulos, et al., 2018; Vouliotis et al., 2013)

Although the pathogenetic background is not totally clarified, supraventricular arrhythmias are rather infrequently recognized as the only underlying condition responsible for syncope. Typical symptoms of supraventricular tachycardia, such as sensation of palpitations, dizziness, or shortness of breath, preceding a syncopal episode, raise the suspicion for a causal link between arrhythmia and loss of consciousness, especially when no signs indicating life‐threatening ventricular tachyarrhythmias have been identified in the diagnostic workup.

Herein, we present a series of four such patients with recurrent syncope episodes with several potential syncope mechanisms revealed on baseline diagnostic approach, thus requiring the documentation of a cause‐and‐effect relationship through an ILR. The recording of supraventricular tachycardia during a recurrent event suggested the optimal treatment approach.

## 2 CASE 1

**FIGURE 1:**
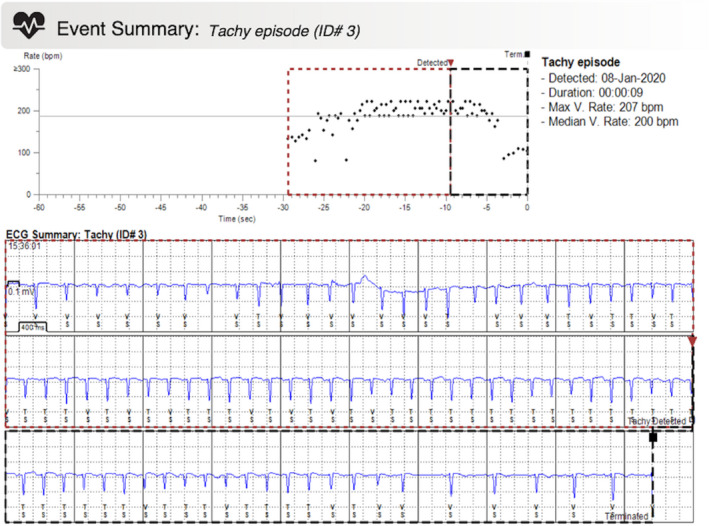
Implantable loop recorder data demonstrating an episode of supraventricular tachycardia at an average rate of 207 bpm, self‐terminating after 9 s, clinically presenting with presyncope

A 41‐year‐old female patient with no past medical history was referred to our outpatient clinic after experiencing recurrent unexplained syncope episodes associated with chest pain and troponin elevation. Her grandfather died suddenly at age of 70 years, while her cousin suffered an acute myocardial infarction at the age of 45 years. She had a normal 12‐lead ECG, while no episodes of tachyarrhythmia, bradycardia, or cardiac pauses were detected in a 24‐hr ECG Holter monitoring with the exception of 374 isolated premature ventricular contractions. There were no late potentials in signal‐averaged electrocardiography (SAECG) neither stenotic lesions found on coronary angiography, while on magnetic resonance imaging (MRI) of the heart, limited fibrosis of probable ischemic pattern with slight left ventricular (LV) dilatation and normal LV contractility was noticed. An electrophysiology study (EPS) revealed the substrate presence for both atrioventricular node reentry tachycardia (AVNRT) and a hemodynamically unstable atrial tachycardia (cycle length 270 ms) that was induced with programmed atrial stimulation, after the intravenous administration of isoproterenol (IVISO). Despite a rather aggressive programmed ventricular stimulation (PVS) protocol from two right ventricular (RV) sites introducing up to three extrastimuli before and after IVISO, no ventricular tachyarrhythmias were induced. A TTT was positive for the induction of neurocardiogenic syncope (NCS). In the presence of multiple syncope mechanisms and in order to resort to the most appropriate and effective treatment modality, a decision to proceed with an ILR was made. Three months after the implantation, the patient presented again with a new presyncope episode associated with palpitations. The analysis of ILR data revealed an episode of supraventricular tachycardia (SVT) at a rate of 207 bpm self‐terminating after 9 s (Figure 1). An ablation procedure was suggested, but the patient declined the offer.

## 3 CASE 2

A 67‐year‐old female patient was referred to our institution for investigation of recurrent unexplained syncope episodes, once resulting in a head injury and other times preceded by prodromal symptoms, mainly dizziness. She had a history of arterial hypertension and dyslipidemia. No abnormalities were detected during a neurological and otolaryngologic examination. She had sinus rhythm on ECG and mild LV hypertrophy with a normal LV ejection fraction in echocardiography with no evidence of organic heart disease. Carotid ultrasonography demonstrated mild atherosclerotic changes in the carotid arteries. A 24‐hr Holter ECG monitoring revealed the presence of frequent premature atrial contractions along with a limited number of supraventricular runs of brief duration in the absence of any bradycardia or cardiac pauses. On EPS, there was evidence of atrioventricular node conduction disease (AVNCD) based on an early appearance of second‐degree AV block on atrial pacing, along with the presence of an AVNRT substrate by revealing dual AVN pathways with the induction of nonsustained runs of AVNRT. No ventricular tachyarrhythmias on PVS were inducible. A mild mixed‐type vagotonic reaction associated with atypical chest pain was observed on TTT. In light of these findings, a treatment with verapamil targeting at the suppression of AVNRT episodes was initiated. In the following 2 years, the patient reported several presyncope episodes, sometimes accompanied by chest discomfort, leading to coronary angiography showing no critical stenosis, before presenting with a new syncope episode resulting in a head injury.

**FIGURE 2:**
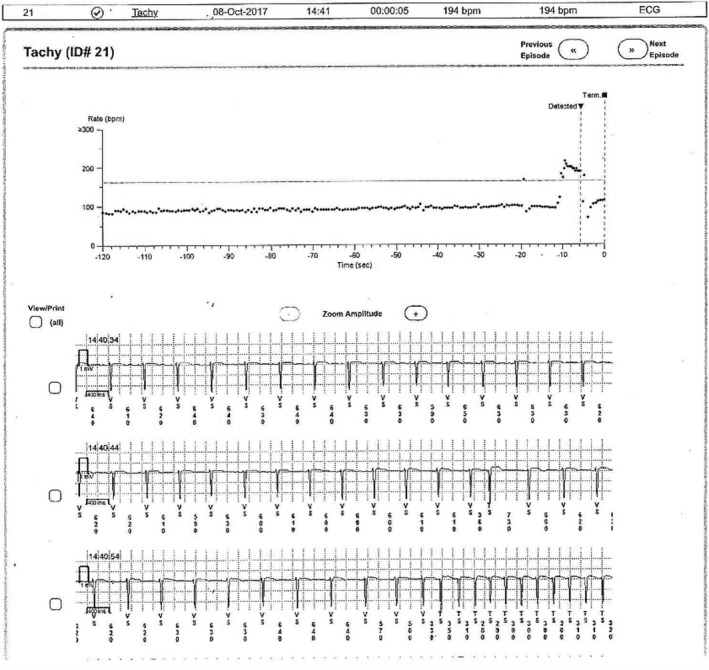
A supraventricular tachycardia episode with an average ventricular rate of 194 bpm and a duration of 5 s detected by an implantable loop recorder, inducing syncope

Once again, facing a patient with multiple potential syncope mechanisms and in order to establish a firm causal relationship, thus establishing the most appropriate treatment plan, namely ablation, pacing, or/and drugs, we decided to proceed with an ILR. Indeed, 6 months later, a symptomatic SVT episode was recorded (Figure 2) and a successful and uncomplicated slow AVN pathway ablation followed. During the 30‐month follow‐up, no recurrent syncope occurred neither any significant tachyarrhythmic nor bradyarrhythmic episodes were recorded.

## 4 CASE 3

A 71‐year‐old man with a 12‐year history of recurrent unexplained syncope episodes in the absence of organic heart disease, occurring once during driving resulting in a car accident and at different times associated with either palpitations or abdominal discomfort, presented with a type II Brugada ECG pattern. After a negative procainamide provocative testing, an EPS and a TTT failed to reveal the syncope mechanism, although short supraventricular tachycardia runs were documented on ambulatory electrocardiography back then. Twelve months ago and after three new syncope episodes, with the concurrent documentation of late potentials on SAECG, a repeat EPS/TTT workup revealed the presence of AVNRT substrate with both induction of the sustained form of the SVT (cycle length 325 ms) and induction of a mixed type of NCS after sublingual nitroglycerine spray provocation.

**FIGURE 3:**
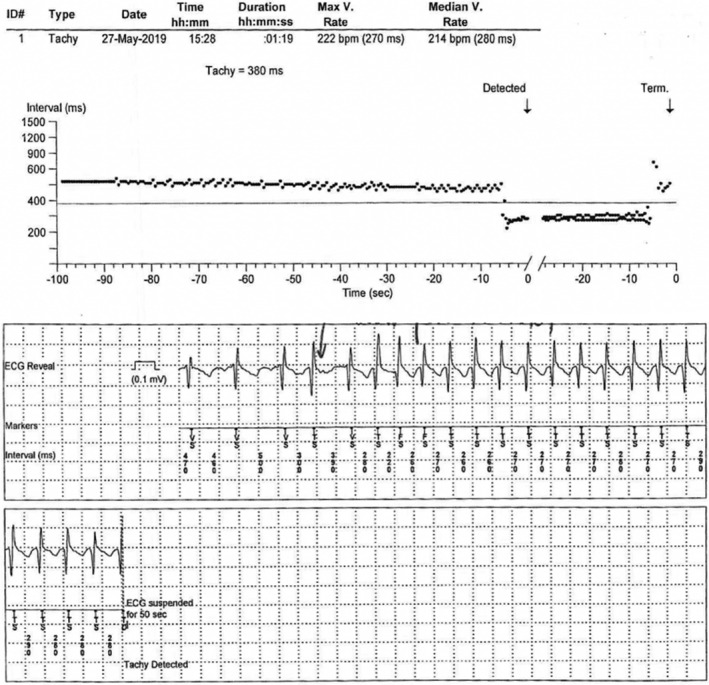
Implantable loop recorder data detecting a supraventricular tachycardia syncopal episode with a median ventricular rate of 214 bpm and a duration of at least 24 s

To further investigate the syncope mechanism, an ILR was offered. A month later, a new syncope episode was associated with an SVT run of 217 bpm detected by the ILR (Figure 3). A successful and uneventful slow AVN pathway ablation was followed 2 weeks later. The patient remains asymptomatic οver the last 12 months.

## 5 CASE 4

A 27‐year‐old male patient with a history of operated Tetralogy of Fallot (TOF) at the age of 4 years presented with recurrent syncope episodes associated with palpitations starting about 14 years after surgical correction. At that time, no hemodynamic abnormalities were detected, while a complete right bundle branch block with a QRS duration of 185 ms was present in the absence of any other significant supraventricular or ventricular ectopy on the 12‐lead ambulatory electrocardiography. On EPS, there was no evidence of sinus or AVN conduction disease. No malignant ventricular tachyarrhythmias were induced after an aggressive PVS protocol. However, after the detection of dual AV node pathway physiology, a sustained form of AVNRT was easily induced on programmed atrial stimulation.

**FIGURE 4:**
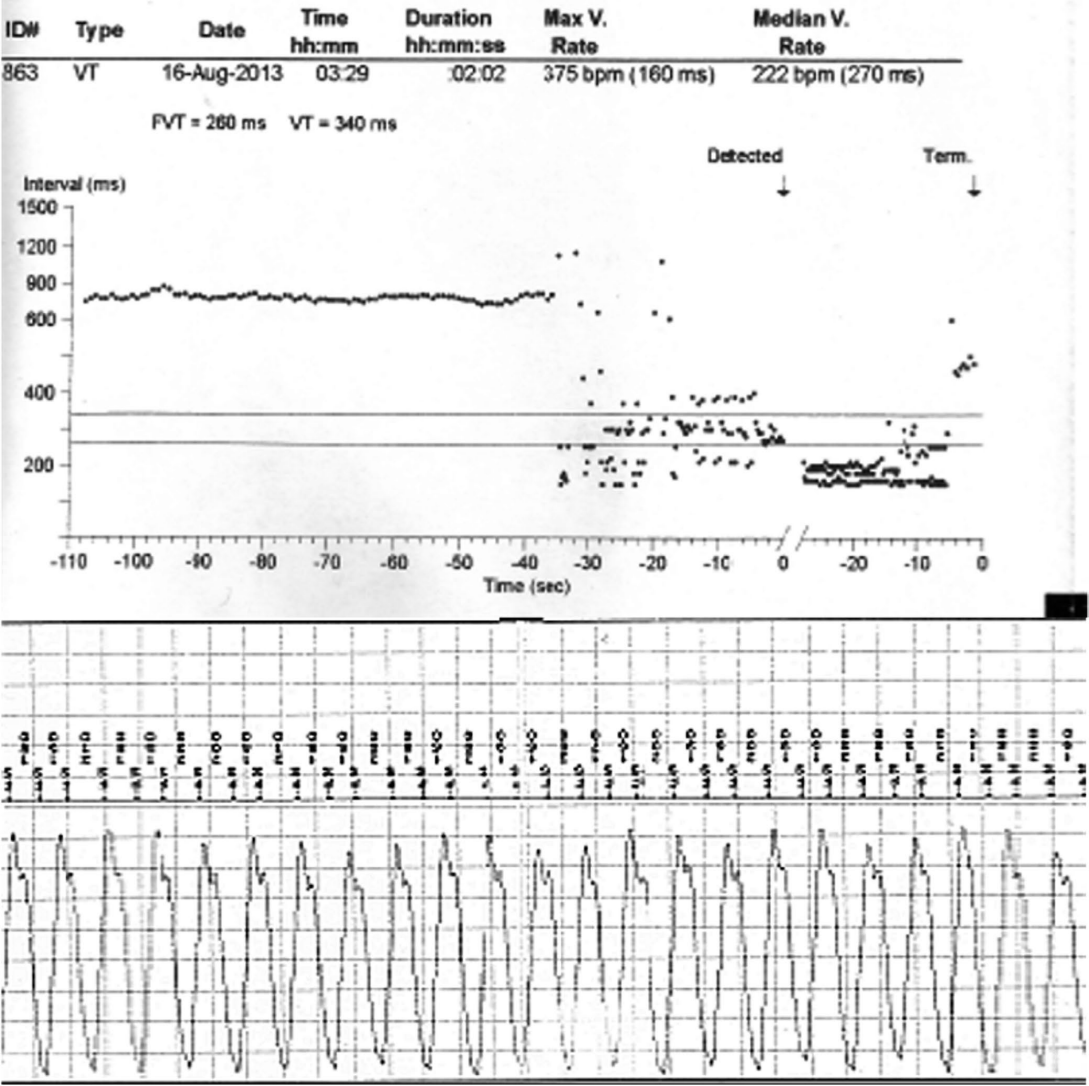
Implantable loop recorder data from a grown‐up congenital heart disease patient showing a regular fast tachycardia run with a median ventricular rate of 222 bpm, self‐terminating after 26 s, associated with syncope

After taken into account the high‐risk patient's profile, a decision was made to proceed with further investigation by long‐term monitoring of an ILR. A new syncope episode occurred 4 months later due to a documented fast tachycardia episode at a rate of 222 bpm (Figure 4). Not being able to rule out the concurrent presence of a nondetected malignant ventricular tachycardia substrate in this operated TOF young patient, we proceeded with an ICD placement at that time. Few months later, an uncomplicated and successful slow AVN pathway ablation took also place. Since then, the patient remains free of any neurological symptoms without any appropriate ICD activations over the last 7 years.

## 6 DISCUSSION

In this case series of four patients presented with rather mixed syncope mechanisms, the ILR was crucial in defining the most appropriate and cost‐effective treatment plan. During the baseline pre‐implantation diagnostic workup of these four patients, more than two potential syncope mechanisms were revealed, namely the presence of at least one SVT substrate with or without evidence for AVNCD and a documented propensity for a NCS reaction in the first three patients. In the fourth Tetralogy of Fallot patient, despite the inability to induce a life‐threatening arrhythmia on an aggressive PVS protocol and in the presence of a well‐documented AVNRT substrate 14 years after the surgical repair, we decided to also proceed with an ILR in order to exclude the probability of sustained ventricular tachycardia as an associated potential cause of syncope in this complicated young grown‐up congenital heart (GUCH) disease patient. Indeed, after detection of a tachycardia‐related recurrent syncope attack on the ILR, we decided to proceed not only with the slow AVN pathway transcatheter ablation, but also with an implantable cardioverter‐defibrillator (ICD) placement.

As in the second and the third cases, the elimination of the slow pathway activity in this fourth GUCH patient resulted in marked symptom improvement with no recurrent syncope attacks and without any need for appropriate ICD activation during the long‐term follow‐up.

The reasons for an SVT like AVNRT to cause transient loss of consciousness have been debated for years. Such a common group of arrhythmias, along with the atrial fibrillation and the atrial flutter commonly met in the general population, cause symptoms such as palpitations, chest discomfort, lightheadedness, and shortness of breath, leading to a significant number of patients to the emergency room.

According to former studies investigating the spectrum of symptoms in patients with SVT, syncope represents a rather uncommon event following tachycardia, more frequently occurring in patients over 65 years of age (Dhala et al., 1995; Kalusche et al., 1998; Wood et al., 1997). However, among older patients, syncope or near‐syncope symptoms are not related to the tachycardia rate, occurring at slower rates than those observed in younger patients (Kalusche et al., 1998).

It is still unclear whether an association between hemodynamic instability, responsible for syncope or presyncope symptoms, and tachycardia rate exists (Yusoff et al., 1988). A heart rate over 170 beats/min was the major factor that was associated with syncope among 167 patients with SVT (Wood et al., 1997). In line with this study and in agreement with the tachycardia rates observed in our patients on ILR, two reports underline that an extremely rapid heart rate during tachycardia is a sufficient condition for the induction of significant hemodynamic changes (Doi et al., 2000; Paul et al., 1990). On the contrary, the induction of high ventricular rate tachyarrhythmia in EPS was well tolerated in Wolff–Parkinson–White patients with a syncope history, suggesting that fast SVT is not necessary a syncope risk factor (Auricchio et al., 1991).

An impaired response of the autonomic vasomotor function to the tachycardia has been proposed as an alternative mechanism for SVT‐associated syncope. Indeed, 3 out of our 4 SVT patients exhibited an abnormal vagotonic reaction on the TTT. Sharing common pathogenetic features with vasovagal syncope, it is assumed that the activation of cardiac mechanoreceptors during tachycardia may lead to a reflex withdrawal of sympathetic tone and an inappropriate stimulation of parasympathetic tone, resulting in vasodepressor mediated syncope (Waxman & Cameron, 1990). High rate SVT leads to an impaired left ventricular filling due to shortening of diastolic time, resulting in a decrease in cardiac output (Inchaustegui et al., 2020). This causes an initial fall in systemic blood pressure followed by a reflex increase in sympathetic tone (Márquez et al., 2016). Sympathetic activation is responsible for further tachycardia acceleration and vigorous ventricular contraction, which, in combination with diminished left ventricular volume, leads to a paradoxical stimulation of left ventricular mechanoreceptors (Iwase et al., 2014). These mechanoreceptors trigger an exaggerated parasympathetic response with further drop of systemic blood pressure, finally resulting in syncope (Gatzoulis & Toutouzas, 2001). Upright position may itself amplify such an inadequate response of the autonomous nervous system due to a greater reduction in left ventricular volume and further enhancement of sympathetic tone (Stewart, 2012). Providing support to the aforementioned hypothesis, Leitch et al. reported no association between syncope during SVT induced on EPS occurring in upright position and the rate of tachycardia (Leitch et al., 1992). Of note, these investigators found a tendency toward a longer cycle length during tachycardia‐induced syncope in the presence of significant hypotension also suggesting parasympathetic stimulation, with both cardioinhibitory and vasodepressor effects, at the time of the event. To further support this hypothesis, a significant association between tachycardia‐induced syncope and a positive TTT for the induction of neurocardiogenic syncope was demonstrated.

In addition to the above mechanisms, several other factors may influence and accelerate the occurrence of SVT‐associated syncope. Among these, patient's behavior at the time of tachycardia seems to play an important role. Immediate cessation of activity after the onset of tachycardia or sensation of prodromal symptoms of syncope and assumption of the supine position may prevent syncope. Furthermore, hypovolemia due to dehydration or blood loss, body position, hypersensitivity of left ventricular wall mechanoreceptors, antiarrhythmic drugs, and any kind of emotional stress may also create the suitable substrate for an SVT‐related syncopal episode (Fenton et al., 2000).

Despite the evolution of novel diagnostic technologies, the identification of the etiology of syncope continues to pose major diagnostic and therapeutic challenges. Initial assessment includes a detailed history, physical examination, a 12‐lead electrocardiogram (ECG), and an echocardiography study to exclude structural heart disease. Ambulatory ECG Holter monitoring is almost routinely used in the investigation of syncope with a rather less diagnostic yield in terms of correlating symptoms with potential arrhythmias (Gibson & Heitzman, 1984; Kühne et al., 2007; Sarasin et al., 2005). A SAECG may reveal late potentials, pointing to the presence of a ventricular reentry mechanism (Gatzoulis, Arsenos, et al., 2018). An EPS in the presence of abnormal 12‐lead, signal‐averaged and ambulatory ECG findings may identify cardiac production and conduction abnormalities or supraventricular and/or ventricular arrhythmias as potential syncope mechanisms (Gatzoulis et al., 2009). In particular, if tachyarrhythmia events are highly suspected, EPS may distinguish a life‐threatening ventricular arrhythmia from an SVT as the underlying syncope cause (Muresan et al., 2019).

For infrequently occurring unexplained recurrent syncope episodes, long‐term monitoring with an ILR is highly recommended, establishing a definite diagnosis in a significant proportion of cases (Edvardsson et al., 2011). In fact, an ILR implantation at an early phase of the diagnostic evaluation of unexplained syncope is preferable and highly recommended by current guidelines, especially in patients with structurally normal hearts, who are considered low risk for malignant ventricular arrhythmias after the initial noninvasive evaluation. In such cases, an EPS could be preserved for further investigation of syncope that remains unexplained or as the final step in the therapeutic management of SVT‐associated syncope combined with the performance of a curative ablation procedure (Brignole et al., 2018). On the contrary, we decided to perform an EPS before proceeding to ILR implantation in the presence of markers suggesting ventricular tachyarrhythmia as the cause of syncope in 3 of our cases, including chest pain at the time of syncope, abnormal findings on cardiac MRI, and a positive family history of sudden cardiac death along with isolated ventricular ectopy on Holter monitoring in case 1, a type II Brugada ECG pattern along with the concurrent presence of ventricular late potentials on SAECG in case 3, and the background of a surgically corrected congenital heart disease in case 4 with a standard QRS duration of 185 ms on the 12‐lead ECG (Gatzoulis et al., 1995), while the reverse of the diagnostic sequence in case 2 with an ILR preceding EPS could be considered reasonable.

There is an ongoing debate about the diagnostic value of the TTT. Exposing the patient to an orthostatic challenge in the upright tilt, the test may unmask autonomic system malfunction responsible for inappropriate vasodepressive and/or cardioinhibitory response (Teodorovich & Swissa, 2016). In addition to this, TTT is recommended as a useful method to predict impaired vasomotor function accompanied by syncope during SVT episodes (Doi et al., 2000).

Our case series demonstrate clearly that mixed syncope mechanisms may coexist in some patients (Dilaveris et al., 2020; Gatzoulis, Georgopoulos, et al., 2018; Vouliotis et al., 2013). This might explain the occasional syncope recurrence in a small proportion of appropriately treated with permanent pacing bradycardia patients (Gatzoulis et al., 1999). It is also well known that vasovagal syncope patients may develop alternative syncope mechanisms during long‐term follow‐up (Gatzoulis et al., 2003). The role of ILR is important in such cases in order to introduce the most effective treatment plan. As shown in our patients with EPS evidence of an underlying SVT substrate, a definite cause‐and‐effect relationship can be established, guiding the appropriate radical ablative treatment. Although doubts are raised about the nature of the tachycardia observed on the ILR during a recurrent syncope episode in our high‐risk GUCH patient, symptoms were also eliminated after the slow pathway ablation in this patient as well, while the concurrently introduced ICD has never been activated so far.

In conclusion, a definite cause of syncope was established by only resorting to an ILR policy in these patients with several syncope mechanisms uncovered during a full‐blown conventional diagnostic strategy, including noninvasive ECG‐related indices and TTT along with a comprehensive EPS. Such a strategy not only confirmed the SVT‐induced syncope mechanism but also led to a curative ablative procedure justifying any associated procedural risks.

## CONFLICT OF INTEREST

The authors declare that they have no conflicts of interest.

## ETHICS

Informed consent for publication of data was obtained from each patient.

## Data Availability

The data that support the findings of this study are available from the corresponding author upon reasonable request.
